# Characterization of Condensed Tannins from Purple Prairie Clover (*Dalea purpurea* Vent.) Conserved as either Freeze-Dried Forage, Sun-Cured Hay or Silage

**DOI:** 10.3390/molecules23030586

**Published:** 2018-03-06

**Authors:** Kai Peng, Qianqian Huang, Zhongjun Xu, Tim A. McAllister, Surya Acharya, Irene Mueller-Harvey, Christopher Drake, Junming Cao, Yanhua Huang, Yuping Sun, Shunxi Wang, Yuxi Wang

**Affiliations:** 1 Key Laboratory of Animal Nutrition and Feed Science (South China) of Ministry of Agriculture, Guangdong Key Laboratory of Animal Breeding and Nutrition, Institute of Animal Science, Guangdong Academy of Agricultural Science, Guangzhou 510640, China; pengkai1016@126.com (K.P.); junmcao@163.com (J.C.); huangyh111@126.com (Y.H.); sunnyxrdragon@sohu.com (Y.S.); 2 Agriculture and Agri-Food Canada, Lethbridge Research and Development Centre, Lethbridge, AB T1J 4B1, Canada; zhongjun.xu@agr.gc.ca (Z.X.); tim.mcallister@agr.gc.ca (T.A.M.); surya.acharya@agr.gc.ca (S.A.); 3 College of Engineering, China Agriculture University, Beijing 100083, China; wsx68@cau.edu.cn; 4 College of Animal Science and Technology, Yangzhou University, Yangzhou 225009, China; huangq0315@126.com; 5 Chemistry and Biochemistry Laboratory, School of Agriculture, Policy and Development, University of Reading, Reading RG6 6AT, UK; i.mueller-harvey@reading.ac.uk (I.M.-H.); chrsdrake@googlemail.com (C.D.)

**Keywords:** tannin composition, purple prairie clover, conservation method, protein precipitation, *Escherichia coli*

## Abstract

Conservation methods have been shown to affect forage nutrient composition and value, but little information is available about the effect of forage conservation on plant condensed tannins (CT). The objective of this study was to assess the effects of conservation method on the concentration, chemical composition and biological activity of CT. Whole-plant purple prairie clover (PPC, *Dalea purpurea* Vent.) was harvested at full flower and conserved as freeze-dried forage (FD), hay (HAY) or silage (SIL). Concentration of CT in conserved PPC was determined by the butanol-HCl-acetone method. Structural composition, protein-precipitation capacity and anti-bacterial activity of CT isolated from conserved forage were determined by in situ thiolytic degradation followed by HPLC-MS analysis, a protein precipitation assay using bovine serum albumin and ribulose 1,5-disphosphate carboxylase as model proteins and by an *Escherichia coli* (*E. coli*) growth test, respectively. Conservation method had no effect on concentration of total CT, but ensiling decreased (*p* < 0.001) extractable CT and increased (*p* < 0.001) protein- and fiber-bound CT. In contrast, hay-making only increased (*p* < 0.01) protein-bound CT. Regardless of conservation method, epigallocatechin (EGC), catechin (C) and epicatechin (EC) were the major flavan-3-ol units, and gallocatechin (GC) was absent from both terminal and extension units of PPC CT. The SIL CT had the lowest (*p* < 0.001) EGC, but the highest (*p* < 0.01) EC in the extension units. Similarly, SIL CT exhibited a lower (*p* < 0.001) mean degree of polymerization (mDP), but higher (*p* < 0.001) procyanidins (PC) than FD or HAY CT. The protein-precipitating capacity of CT in conserved PPC ranked (*p* < 0.001) as FD > HAY > SIL. *E. coli* growth n M9 medium was inhibited by 25–100 µg/mL of CT isolated from FD, HAY and SIL (*p* < 0.05), but preservation method had no effect on the ability of CT to inhibit bacterial growth. The results demonstrated that ensiling decreased the extractability and protein-precipitating capacity of CT by increasing the proportions of PC. Purple prairie clover conserved as hay retained more biologically active CT than if it was conserved as silage.

## 1. Introduction

Condensed tannins (CT) are a group of naturally occurring phenolic compounds that are widely present in plants including a number of common forages. Condensed tannins are oligomeric or polymeric flavonoids consisting of flavan-3-ol units that commonly include catechin (C), epicatechin (EC), gallocatechin (GC) and epigallocatechin (EGC) with the relative proportions of these flavonoids differing among plant types. Condensed tannins exhibit various antimicrobial, anti-parasitic, anti-oxidant, and anti-inflammatory activities and as a result are seen as a promising natural alternative to in-feed antibiotics [1]. It has been shown that concentration and composition of CT in a plant are affected by the growing conditions, phenological growth stage as well as tissue type [2,3,4,5]. Ensiling and hay-making are two common methods used to conserve forage as silage or hay for ruminant livestock. It is generally recognized that these conservation methods alter the nutrient composition of forage, leading to changes in the feed value of conserved forages [6]. For example, excessive proteolysis can occur during ensiling, decreasing protein nutritive value [7]. Although studies have found that forage conservation methods also reduce the extractability of CT [6], their effects on the structural nature and specific biological activity of CT have not been examined. Research in this area is needed because the biological activities of CT in forage are closely associated with their concentrations and chemical structures [8].

Purple prairie clover (PPC; *Dalea purpurea* Vent.) is a native legume that is widely distributed in the North America prairie and contains high concentrations of CT (up to 94 g/kg DM). It has been shown that CT in PPC possess strong anti-*Escherichia coli* activity [9,10,11,12]. In addition, PPC CT cause greater precipitation of bovine serum albumin (BSA) and spinach ribulose 1,5-disphosphate carboxylase (Rubisco) [5,9] than CT from other plant sources. Huang et al. [6] suggested that the biological activity of CT in PPC conserved as hay was higher than that in silage because more of the CT remained in an extractable form. However, the chemical composition of the extractable CT in these conserved forages was not determined. The objective of this study was to assess the effects of PPC conservation method on the concentration, chemical composition and biological activity of CT.

## 2. Results

### 2.1. Characteristics of PPC Conserved as FD, HAY and SIL

Although organic matter (OM) and crude protein (CP) were not affected by forage conservation method (Table 1), PPC conserved as FD had lower (*p* < 0.01) concentrations of neutral detergent fibre (NDF) and acid detergent fibre (ADF) than HAY or SIL.

The acid detergent lignin (ADL) content of FD was lower (*p* < 0.01) than HAY. Contents of NDF, ADF and ADL were similar (*p* > 0.05) between HAY and SIL. The water-soluble carbohydrate (WSC) concentration in conserved forages ranked as FD > HAY > SIL (*p* < 0.001). Among the conserved forages, SIL contained the lowest (*p* < 0.001) level of total phenolics, followed by HAY and FD, respectively. Similarly, SIL exhibited lower (*p* < 0.001) extractable CT than FD or HAY. However, SIL had greater (*p* < 0.001) concentrations of fiber-bound and protein-bound CT than FD or HAY. Protein-bound CT were also greater (*p* < 0.001) in HAY than in FD. Conservation method had no effect on the concentration of total CT (*p* > 0.05).

### 2.2. CT Terminal and Extension Units

Regardless of conservation method, the terminal units of PPC CT were composed of C and EC only, and no GC or EGC was detected.

**Figure 1:**
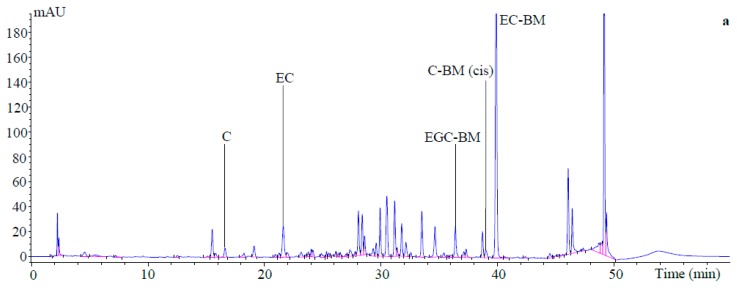

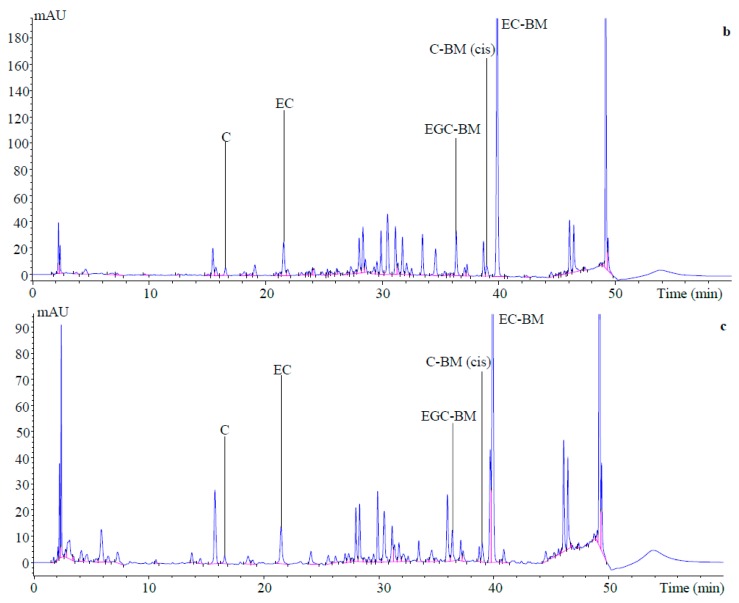
HPLC chromatograms (280 nm) of thiolysis reaction products that were obtained from (**a**) freeze-dried; (**b**) hay and (**c**) silage of whole-plant purple prairie clover. Terminal units: C = catechin, EC = epicatechin; Extension units as BM-adducts: EGC-BM = epigallocatechin, C-BM = catechin, EC-BM = epicatechin, where BM = benzyl mercaptan.

Following thiolytic degradation, two flavan-3-ols (i.e., C and EC), were detected in terminal units as well as EGC-benzyl mercaptan (BM), C-BM and EC-BM adducts in extension units in all samples, regardless of conservation method (Figure 1). Terminal units accounted on average for 5–8% of all flavan-3-ol units in tannins. The extension units (92–95%) contained three flavanols, i.e., EGC (19–28%), C (2–4%) and EC (64–69%), but lacked GC (Table 2).

Condensed tannins present in HAY had greater (*p* < 0.001) proportion of EGC, but lower (*p* < 0.01) EC in the extension units than FD and SIL. In contrast, CT from SIL had lower (*p* < 0.001) EGC, but a greater (*p* < 0.001) proportion of C as compared to FD.

The average polymer size of CT, ranged from 12.4 to 18.6 of mean degree polymerization (mDP) in conserved PPC (Table 3). The mDP was lower (*p* < 0.001) in SIL as compared to FD or HAY.

Similarly, CT in SIL had a lower (*p* < 0.001) proportion of prodelphinidins (PD; i.e., GC + EGC), but a higher (*p* < 0.001) proportion of procyanidins (PC; i.e., C + EC) than FD and HAY. In contrast, CT in HAY had a higher (*p* < 0.001) proportion of PD but lower (*p* < 0.001) proportion of PC than FD PPC. Irrespective of conservation method, PPC CT were dominated by the *cis*-isomers (i.e., EC + EGC). SIL had a lower (*p* < 0.001) proportion of *cis*-isomers and higher (*p* < 0.001) proportion of *trans*-isomers than in FD or HAY. There was no difference (*p* > 0.05) in *cis-* and *trans-i*somers between FD and HAY.

### 2.3. Protein-Precipitating Capacities of CT in PPC Conserved as FD, HAY and SIL

**Figure 2:**
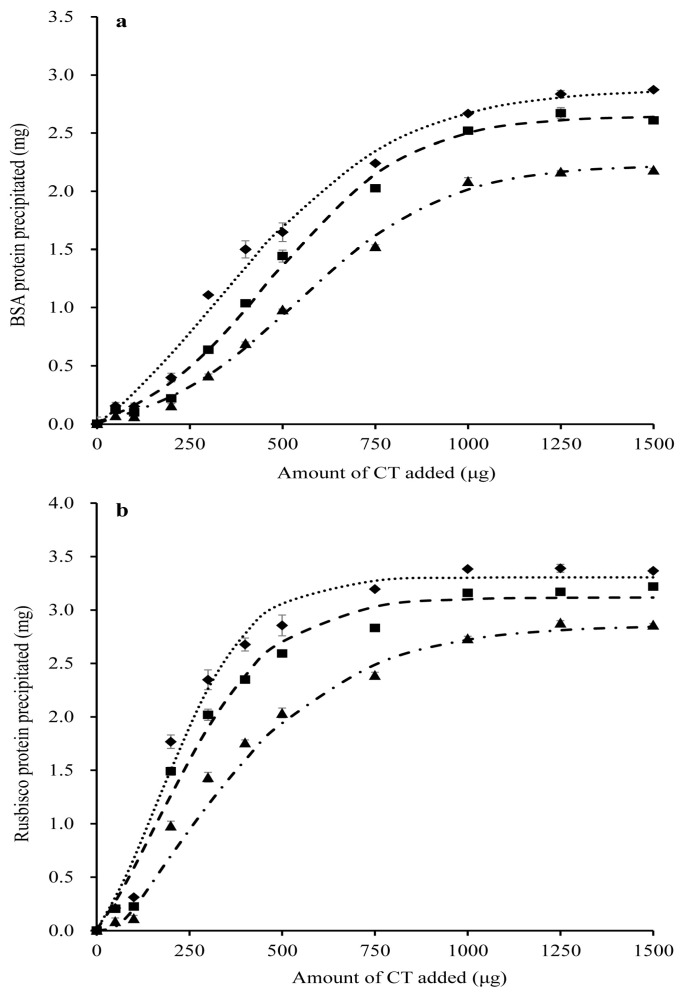
Precipitation of bovine serum albumin (BSA; **a**) and spinach ribulose 1,5-disphosphate carboxylase (Rubisco; **b**) by condensed tannins (CT) isolated from freeze-dried purple prairie clover (♦FD), or conserved as hay (■HAY) or silage (▲SIL). The dash lines relate to corresponding CT data which were fitted to a SigmaPlot curve as described under statistical analysis. Bars indicate standard error and where not visible, fall within symbols.

Bovine serum albumin was completely precipitated by ≥1000 μg PPC CT (Figure 2a), whereas Rubisco was completely precipitated by ≥750 μg PPC CT (Figure 2b). The protein-precipitating capacity of CT in the conserved PPC ranked in the order of FD > HAY > SIL (*p* < 0.001) for both BSA and Rubisco proteins. Condensed tannins from PPC, consistently exhibited a lower (*p* < 0.001) ability to precipitate BSA than Rubisco, irrespective of conservation method (Table 4).

### 2.4. Growth of E. coli Affected by CT in PPC Conserved as FD, HAY and SIL

Compared to the control and irrespective of conservation method, growth of *E. coli* 25922 and 35281 was inhibited (*p* < 0.001) at all concentrations of CT tested (Table 5). The maximal growth rate (*µ*) of strain *E. coli* 25922 decreased (*p* < 0.001) as the CT increased from 25 to 100 µg/mL, whereas no difference was observed at CT < 100 µg/mL for *E. coli* 35281. Lag times (*L*) increased with increasing CT concentrations for both *E. coli* 25922 (*p* < 0.001) and 35281 (*p* < 0.05).

The maximal growth rate of *E. coli* 25922 did not differ among conservation methods with the exception of CT at 25 µg/mL. Strain *E. coli* 25922 exposed to 25 µg/mL of FD CT had a greater (*p* < 0.01) *L*, but a lower (*p* < 0.01) *µ* than that exposed to HAY or SIL CT. In contrast, *µ* and the *L* of *E. coli* 35218 were not affected by the source or concentration of CT, excepting that 100 µg/mL of CT from HAY and SIL decreased (*p* < 0.05) *L* as compared to CT from FD.

## 3. Discussion

### 3.1. Effect of Conservation Method on the Compositions of PPC

The chemical composition of PPC forage conserved as FD, HAY and SIL was similar to the report by Huang et al. [6]. The concentrations of extractable, protein-bound and fiber-bound CT were also comparable with previous reports [6,13,14]. In this study, the higher NDF concentration in HAY and SIL compared with FD suggests that hay-making or ensiling increased the fibre concentration of PPC. The increased NDF in SIL reflects the microbial fermentation of soluble nutrients during ensiling as shown by the lower WSC content of SIL PPC compared to FD PPC. A significant increase in the post-ensiling NDF content of alfalfa was also observed by Broderick [15]. Although the increase of NDF in HAY may also reflect plant and microbial utilization of WSC during drying and post-baling, it is also a result of the loss of leaves, which lowers the leaf-to-stem ratio. As stems contain more NDF than leaves, it also increases the NDF content of the conserved forage [16]. Loss of leaves during hay-making is one of the major factors that contribute to differences in the nutritive value between hay and other forage conservation methods [17]. Enoh et al. [18] also reported that hay-making increased NDF content of *Brachiaria* as compared to green forage.

Similar concentration of extractable CT in HAY and FD PPC suggests that sun-drying had minimal effect on biological activity of CT in PPC. Similar results were also reported for PPC [6] and sainfoin (*Onobrychis viciifolia* Scop.) hay [19,20,21]. On the contrary, other studies have observed that extractable CT in sun-cured sericea lespedeza (*Lespedeza cuneate* (Dum. Cours.) G. Don) and sainfoin hay was lower than that in the fresh forage [22,23]. The discrepancy among these studies is likely due to variations in drying conditions and forage type. Scharenberg et al. [20] dried sainfoin in a closed system at 30 °C, while Lorenz et al. [21] wilted sainfoin to a moisture level of 50%, while the PPC in the present study was wilted in the field under hot and windy conditions.

It is interesting to note that ensiling reduced extractable, but increased protein-bound and fiber-bound CT, with no effect on total CT. This result indicates that a portion of extractable CT has been transformed into protein-bound and fiber-bound during ensiling. A shift from extractable to bound CT during the ensiling of PPC was also observed by Huang et al. [6]. It is likely that partial disruption of plant cells as a result of physical chopping before ensiling and microbial fermentation during ensiling enables CT to react with other plant fractions, increasing the bound CT fraction [24]. Ensiling sainfoin (*Onobrychis viciifolia* Scop.) also decreases extractable and increases bound CT [19,20]. Because biological activity of CT in the plant depends on both chemical structure as well as the concentration of the extractable CT [8,25], the reduced extractability of CT in PPC silage suggests that this is the primary factor responsible for the reduced biological activity of PPC CT.

### 3.2. Effects of Forage Conservation Method on the Structure and Chemical Composition of PPC CT

Although it has been found that conservation methods affect the concentration of CT in forage [6,24], there is no information available about the effects of forage conservation on the chemical structure of CT. To our knowledge, this is the first study that demonstrates that ensiling and hay-making alters the structural characteristics of CT in conserved forage.

The flavan-3-ol composition of PPC in this study was consistent with that of CT extracted from various phenological PPC tissues, with epicatechin (average 72.2%) and epigallocatechin (average 24.2%) being the dominant monomers and gallocatechin being noticably absent from both terminal and extension units [5]. This is comparable to *Lotus corniculatus* CT, which contained about 67% epicatechin and lacked gallocatechin in both terminal and extension units [26,27]. However, the structural composition of CT in PPC also differs from some other forage sources. For examples, epigallocatechin was the principal monomer (≈64%) in *Lotus pedunculatus* [27] and sainfoin CT (52–63%) [28]. In addition, PPC CT in the present study contained more PC than PD, which was consistent with our previous study with PPC [5] but differing from sainfoin CT, which contain more PD than PC [28,29,30,31]. Consistent with most sources of CT, both sainfoin and PPC CT contained more *cis* than *trans* units. These results indicate that the structural composition of CT are plant species specific, which probably also contributes to the varying biological activity of different CT.

Compared to altering the extractability of CT, forage conservation method had a relatively minor impact on the structural composition of CT in PPC. Those minor alterations that were noticeable included an increase in PC with ensiling and PD with hay-making. Ensiling apparently decreased mDP and the proportion of *cis*-isomers, whereas hay-making did not affect these parameters. This suggests that ensiling had a greater effect on the composition of CT than hay-making. The reason that ensiling decreased mDP are unknown, but may be attributed to the higher proportion of PC in the ensiled PPC. It has been reported that CT with higher PC tend to have lower mDP and lower extractable CT concentrations [32,33,34]. Vidal et al. [35] and Tharayil et al. [36] also observed that mild acidic conditions could cleave interflavanic bonds, reducing the degree of polymerization of CTs. In the present study, the pH of PPC declined to 4.8 after one week and remained at this level throughout 10 weeks of storage. Therefore, the mild acidic conditions of the ensiling process may have contributed to a decline in mDP of CT in silage. Whether microbial activity during ensiling had any effect on this response is unknown, although there are significant CT-microbe interactions during ensiling [37]. As compared with ensiling, results indicated that hay-making by sun-curing under the conditions in this study had little effect on CT composition as demonstrated by the similar mDP and proportions of *cis*- and *trans*-isomers of FD and HAY samples.

### 3.3. Effect of Forage Conservation Method on the Protein-Precipitating Capacity of PPC CT

Previous studies have reported that the protein-precipitating capacities of PPC CT in different phenological tissues of differing maturity is a reflection of the chemical composition of CT [5]. The fact that the protein-precipitating capacity of CT decreased in the order of FD > HAY > SIL indicates that both ensiling and hay-making decreased protein-precipitating capacity, with this response being greater for silage. Binding and precipitating proteins is a common characteristic of CT and is the principal factor responsible for their biological activity [38]. The capacity of CT to bind proteins is determined by their chemical traits including chain length, molecular weight, PC/PD ratio, number of potential hydrogen and hydrophobic bonding sites and conformation [26,39,40]. Therefore, the decreased protein-precipitating capacity of CT by hay-making and ensiling in this study is likely due to alterations in their chemical structure. It has been demonstrated that the affinity of CT for proteins decreases with decreasing mDP [41,42,43,44,45] and proportions of PD [46,47,48]. Moreover, De Freitas and Mateus [49] found that the number of active sites that were able to bind proteins increased with the number of *cis*-flavanol units. Therefore, the decreased protein-precipitating capacity of CT after ensiling is likely attributable to the decline in mDP, proportions of PD and cis-flavanol units. However, the decrease in the protein-precipitating capacity of CT in HAY as compared to FD PPC cannot be explained by these factors as they did not differ between these two methods of forage conservation. This suggests that the protein-binding capacity may not only be related to mDP or the proportions of PD proportion or *cis*-flavanol units, but also due to the comprehensive effect of flavanol units or the spatial distribution of these monomers within CT [50]. Additional studies have indicated that CT with higher molecular weight (MW) exhibit stronger protein-binding capacities [51,52,53,54] as CT with higher MW contain a larger number of hydroxyl groups that promote the formation of cross-links with proteins [55]. It is also possible that the lower protein-precipitating capacities of ensiled CTs as compared to those from hay may reflect a reduction in the MW of CT during ensiling, but this was not determined in this study. Further research is needed to confirm this.

### 3.4. Effect of Forage Conservation Method on Anti-E. coli Activity of PPC CT

Condensed tannins in PPC have been shown to possess strong anti-*E. coli* activity both in vitro [9,10,56] and in vivo [12,13]. Results of the present study were consistent with these previous observations. Furthermore, this study demonstrated that although ensiling and hay-making altered the structural composition and protein-precipitating capacity to a degree, they did not affect the anti-*E. coli* activity of the PPC CT. This suggests that in addition to protein-precipitating ability, other factors may also be responsible for the involved in anti-*E. coli* activity of PPC CT. This is conceivable given the fact that protein-precipitation is a universal property of CT, but only CT from certain plant sources (e.g., *Acacia catechu*, *Holarrhena antidysenterica*, *Quercus infectoria*, *Uncaria gambir*, *Walsura robusta, Vaccinium macrocarpon,* PPC) have known anti-*E. coli* activity [10,57,58,59,60,61,62]. Liu et al. [9] reported that the anti-*E. coli* activity of PPC CT is attributable to their high affinity for proteins and ability to cause cell aggregation. This destabilizes the bacterial outer membrane (OM) and commensurately causes an alteration in its fatty acid composition [10]. Flavan-3-ol monomers, such as catechins in green tea (*Camellia sinensis*), have also been reported to express anti-*E. coli* activity by directly binding peptides of bacterial origin [63], damaging the liquid bilayer and increasing OM permeability [64,65]. It should be noted that although anti-*E. coli* actity of CT isolated from SIL and HAY did not differ to that from FD PPC, the actual anti-*E. coli* activity would be reduced because of a reduction in the extractibility of CT from the whole plant. Conservation method, especially ensiling, decreased the extractability of CT. Therefore further study is needed to elucidate the mechanisms by which forage conservation method affect biological activity of CT.

## 4. Materials and Methods

### 4.1. Conservation of PPC as Freeze-Dried, Hay or Silage

Whole-plant PPC was harvested at full-bloom from three irrigated plots (approximately 0.25 acre each) of the Swinton silt loam soil (Orthic Brown Chernozem) [66] at the Lethbridge Research and Development Centre (Lethbridge, AB, Canada). Harvested forage from each plot was divided into three equal lots to be conserved as FD, HAY, or SIL. The lot for FD was frozen at −40 *°*C immediately after harvest and then lyophilized. The FD was used to simulate green chopped forage and therefore its CT content was considered to represent that of freshly harvested forage. HAY was allowed to sun cure in the field to <15% moisture, and was subsequently baled in approximately 20-kg square bales (90 × 50 × 30 cm; 3 bales) and stored in an enclosed shed at ambient temperature for 120 d. Subsamples from at least 3 locations of each bales were collected, composited and lyophilized. Forage for SIL was wilted in the field to ≈30% DM and chopped to a theoretical chop length of 1.0 cm using a paper cutter (X-ACTO 26612, Westerville, OH, USA). Chopped PPC was then packed (≈2.7 kg) into three PVC laboratory silos (10 × 35.5 cm) and compacted with a hydraulic press to achieve a packing density of approximately 890 kg (fresh weight)/m^3^. Silos were sealed at both ends with rubber lids and metal bands, with one lid fitted with a 7.0-cm-long rubber tube as a vent. Laboratory silos were stored indoors at 22 °C and were opened after 76 d of ensiling. Upon opening, silage within 5.0 cm at both ends was discarded, and the remaining content of each silo was thoroughly hand mixed and subsamples taken. The subsamples from the three silos of each plot were composited, frozen at −40 °C and immediately lyophilized.

All forage samples were ground to pass 1.0 mm screen using a Thomas Wiley Cutting Mill (Arthur H. Thomas Co., Philadelphia, PA, USA) and stored in amber glass containers for chemical analyses. Subsamples of each type of preserved forages were also composited across the three plots to form a single sample for CT extraction, estimation of protein precipitating capacity and anti-*E. coli* activity.

### 4.2. Chemical Analysis

Conserved forage samples were analyzed for DM, OM and acid detergent lignin using AOAC method [67], NDF and ADF using an ANKOM 200 system (ANKOM Technology Corp., Fairport, NY, USA) with sodium sulfite and α-amylase added for NDF analysis. Samples were ball-ground in a planetary micro mill (Retsch Inc., Newtown, PA, USA) for measurement of total nitrogen (N) by flash combustion analysis using a NA1500 Nitrogen Analyzer (Carlo Erba Instruments, Milan, Italy). For WSC, 15 g subsamples from conserved forage were combined with 135 g of deionized H_2_O and blended in a homogenizer (Osterizer, Sunbeam, Fontana, CA, USA) for 30 s. The homogenate was strained through four layers of cheesecloth and the supernatant was sampled and analyzed for WSC as described by Zahiroddini et al. [68]. Conserved PPC samples were analyzed for total phenolic compounds by the Folin-Ciocalteu method [69] with tannic acid (Sigma, St. Louis, MO, USA) as the standard and were expressed as tannic acid equivalents. The concentrations of extractable, protein-bound and fibre-bound CT were determined using the method of Terrill et al. [70] with CT purified from whole PPC plants as a standard.

### 4.3. In Situ Thiolysis of PPC Tannins and HPLC Analysis of Thiolysis Products

Condensed tannins were degraded with BM as described previously [31]. The resulting flavan-3-ols (terminal subunits) and their BM-adducts (extension subunits) were identified by HPLC-MS analysis [71,72] and quantified using peak areas at 280 nm in conjunction with published flavan-3-ol response factors against taxifolin [31,73]. This yielded data on the mean degree of CT polymerization, molar percentages of PC and PD within CT, and molar percentages of *cis*- and *trans*-flavan-3-ols [73].

### 4.4. Determination of the Protein Precipitation Capacities of CT from Freeze-Dried, Silage or Hay PPC

Condensed tannins from PPC conserved as FD, HAY and SIL were extracted and purified as described by Wang et al. [74]. The purified CT were stored in a sealed amber glass bottle at −20 °C and the same batch of each CT were used in all assays. The protein precipitation capacities of these purified CT were determined using a modified precedure described by McAllister et al. [75]. Bovine serum albumin and Rubisco (MW 557) from spinach (Sigma-Aldrich) were used as model proteins for determining the relative capacities of the extracted CT to bind protein. The BSA was dissolved (3 mg/mL) in 0.2 M acetate buffer (pH = 5.0) containing 0.17 M NaCl, and the Rubisco was dissolved (4 mg/mL) in 1 M 2-amino-2-(hydroxymethyl)-1, 3-propanediol hydrochloride (Tris HCl; pH = 7.8). One milliliter of each protein solution was combined with 0.5 mL of aqueous solutions containing 0, 50, 100, 200, 300, 400, 500, 750, 1000, 1250 or 1500 µg of CT from each source. Each mixture was vortexed, allowed to stand at room temperature for 30 min and centrifuged (15,600× *g*, 10 min). The CT remaining in a 1-mL subsample of supernatant were removed by adding 0.5 mL of aqueous polyethylene glycol (Sigma-Aldrich; MW 6000; 12 mg/mL) followed by centrifugation. Protein remaining in solution was quantified colorimetrically (OD_595_) using a Dye Reagent Concentrate Kit (BioRad Laboratories, Mississauga, ON, Canada) against an original freshly prepared solution of BSA or Rubisco protein as a standard. Each assay consisted of six replicates for each dose of CT and the assay was repeated three times over a 1-week period.

The amount of protein precipitated was calculated as the difference between added protein and that present in the supernatant after CT addition. Data were fitted to a sigmoidal curve using nonlinear regression in SigmaPlot for Windows (version 13.0; Systat Software Inc., Santa Jose, CA, USA):

y = a0 + a/(1 + exp(−(x − b)/c))

where *y* = mg of protein (BSA or Rubisco) precipitated, *x* = μg of CT incubated, *a*_0_ + *a* = estimated maximum amount (mg) of protein (BSA or Rubisco) precipitated, *b* = sigmoidal centre (mg of CT at the 50% of maximal protein precipitation), and *c* = sigmoidal width. The protein-precipitating capacity (*PP*) of each CT was expressed as the amount (μg) of the CT required to precipitate 1.0 mg of BSA or Rubisco protein.

### 4.5. Determination of CT from Freeze-Dried, Silage or Hay PPC on the Growth of E. coli

Two generic *E. coli* strains (ATCC 25922 and ATCC 35281) were obtained from the Lethbridge Research and Development Centre culture collection. Both strains of bacteria were grown at 37 °C with shaking (175 rpm, 16 h) for pre-incubation prior to being used as inoculants. An in vitro pure culture experiment was conducted using glass tubes (13 × 100 mm) with M9 medium including (g/L) M9 minimal salt (11.28), casamino acids (5), glucose (4.5), MgSO_4_·7H_2_O (0.239) and CaCl_2_ (0.011). Prepared M9 medium (500 mL) was transferred into 1.0-L serum vials and sterilized by autoclaving. After cooling, each of pre-incubated bacterial cultures (30 µL) were added to a 200 mL of M9 medium and cultures were adjusted to identical densities (OD_600_ value was approximately 0.01) by diluting with M9 medium. Immediately after inoculation, 30 µL of filter-sterilized solutions of each purified CT (FD, HAY and SIL) at the concentrations of 0, 2, 500, 5000 and 10,000 µg/mL were added to the bacterial culture (3 mL), yielding 0, 25, 50 or 100 µg CT/mL in inoculated cultures. Triplicate vials were prepared for each bacterial strain and CT concentration. Triplicate vials for each CT concentration, but without bacterial inoculum were also prepared as blank controls. All glass tubes were incubated aerobically with shaking (175 rpm) at 37 °C and assessed for bacterial growth after 0, 4, 6, 8, 12 and 24 h of incubation by measuring optical density at 600 nm (UltraSpec Plus 4054; Pharmacia, Baie, QC, Canada). Optical densities were corrected for the blank controls and the culture was diluted with M9 medium so that all OD_600_ were within the range of 0–0.5. The OD_600_ values were then fitted to the following modified Gompertz equation [76]:

In (ODt/OD0) = A×exp {−exp[(μ×e/A)×(L−t) + 1]}

where *A* is the logarithmic increase of bacterial population, µ is maximum growth rate (per hour), *L* is the lag time, *t* is the time (in hours), and OD_t_ and OD_0_ are the optical densities obtained at times *t* and zero, respectively.

### 4.6. Statistical Analysis

All data were subjected to analysis of variance as a completely randomized design using the PROC MIXED procedure of SAS [77]. Conservation method was the fix effect for all determinations and plot where the original forage was obtained was the random effect for chemical and CT structural data analysis, whereas repeated run was the random effect for protein precipitating capacity data analysis. Data from *E. coli* strains were originally analyzed as a 3 × 4 factorial design with individual glass tube as a random factor. This revealed a CT level × conservation method interaction over the incubation period. Therefore, data were re-analyzed as a randomized complete block design for each conservation method of each *E. coli* strain. When CT level or CT level × conservation method interactions were significant (i.e., *p* < 0.05), means of the conservation methods were compared at each CT level. Differences among treatments were determined using LSMEANS with PDIFF function and adjusted by a Tukey’s test in SAS with significance declared at *p* < 0.05.

## 5. Conclusions

Ensiling and hay-making both impacted CT extractability, structural composition and protein-precipitating capacity with the effect being greater for silage than for hay. Ensiling decreased the extractability of CT and reduced *cis*-isomers, mDP and protein-precipitating capacity of extractable CT. However, conservation methods did not affect the anti-*E. coli* activity of CT isolated from conserved forage. Overall, purple prairie clover preserved as hay conserved the biological activity of CT via preserving extractable CT more than silage.

## Figures and Tables

**Table 1:** Chemical composition (g/kg DM) of whole-plant purple prairie clover conserved as freeze-dried forage (FD), hay (HAY) or silage (SIL) (*n* = 3).

Item	FD	HAY	SIL	SEM	*p*-Value
Organic matter	924	923	913	3.96	0.174
Crude protein (N × 6.25)	158	157	165	2.20	0.058
Neutral detergent fibre	439 ^b^	497 ^a^	480 ^a^	5.74	0.001
Acid detergent fibre	405 ^b^	422 ^a^	417 ^a^	2.38	0.006
Acid detergent lignin	79.8 ^b^	98.3 ^a^	90.1 ^a,b^	2.79	0.009
Water-soluble carbohydrate	25.4 ^a^	11.5 ^b^	2.4 ^c^	0.34	<0.001
Total phenols ^1^	66.2 ^a^	51.4 ^b^	28.6 ^c^	0.95	<0.001
Condensed tannins (CT)					
Extractable CT	70.2 ^a^	64.1 ^a^	27.4 ^b^	1.53	<0.001
Fibre-bound CT	5.2 ^b^	5.6 ^b^	7.1 ^a^	0.17	<0.001
Protein-bound CT	9.0 ^c^	12.4 ^b^	44.3 ^a^	0.17	<0.001
Total CT	84.5	82.2	78.9	1.45	0.083

^1^ Determined as tannic acid equivalent. ^a, b, c^ Means with different letters differ within rows (*p* < 0.05). SEM, standard error of the mean.

**Table 2:** Flavan-3-ol composition (% molar percentages) in condensed tannins of whole-plant purple prairie clover conserved as freeze-dried forage (FD), hay (HAY) or silage (SIL).

	Terminal Units (%)				Extension Units (%)			
	GC ^1^	EGC	C	EC	GC-BM ^2^	EGC-BM	C-BM	EC-BM
FD	0	0	1.56 ^a^	4.26 ^b^	0	23.50 ^b^	2.42 ^b^	68.26 ^a^
HAY	0	0	1.26 ^b^	4.13 ^b^	0	27.97 ^a^	2.19 ^b^	64.45 ^b^
SIL	0	0	1.29 ^b^	6.94 ^a^	0	19.17 ^c^	3.78 ^a^	68.82 ^a^
SEM	-	-	0.063	0.282	-	0.928	0.164	0.743
*p*-value	-	-	0.008	<0.001	-	<0.001	<0.001	0.002

^1^ GC = gallocatechin, EGC = epigallocatechin, C = catechin, EC = epicatechin. ^2^ Extension units as BM-adducts: EGC-BM = epigallocatechin, C-BM = catechin, EC-BM = epicatechin, where BM = benzyl mercaptan. ^a, b, c^ Means with different letters differ within columns (*p* < 0.05). SEM, standard error of the mean.

**Table 3:** Structural composition of condensed tannins from whole-plant purple prairie clover conserved as freeze-dried forage (FD), hay (HAY) or silage (SIL).

	mDP ^1^	PC (%) ^2^	PD (%)	*Cis* (%) ^3^	*Trans* (%)
FD	17.3 ^a^	76.5 ^b^	23.5 ^b^	96.0 ^a^	4.0 ^b^
HAY	18.6 ^a^	72.0 ^c^	28.0 ^a^	96.6 ^a^	3.4 ^b^
SIL	12.4 ^b^	80.8 ^a^	19.2 ^c^	94.9 ^b^	5.1 ^a^
SEM	0.66	0.93	0.93	0.20	0.20
*p*-value	<0.001	<0.001	<0.001	<0.001	<0.001

^1^ mDP = mean degree of polymerization; ^2^ PC = procyanidin tannins (C + EC); PD = prodelphinidin tannins (GC + EGC); ^3^ *cis* = *cis* isomers of flavan-3-ol subunits (EC + EGC); *trans* = *trans* isomers of flavan-3-ol subunits (C + GC); ^a, b, c^ Means in a column with different letters differ within columns (*p* < 0.05). SEM, standard error of the mean.

**Table 4:** Precipitating capacities of condensed tannins from purple prairie clover conserved as freeze-dried forage (FD), hay (HAY) or silage (SIL).

Protein ^1^	Parameters ^2^	FD	HAY	SIL	SEM	*p*-Value
BSA	*a_0_ + a*	3.1 ^a^	2.9 ^b^	2.4 ^c^	0.01	<0.001
	*b*	323 ^b^	467 ^a^	541 ^a^	0.01	<0.001
	*c*	244 ^a^	185 ^b^	200 ^b^	7.72	0.004
	*PP*	124 ^c^	172 ^b^	223 ^a^	2.57	<0.001
Rubisco	*a_0_ + a*	3.4 ^a^	3.3 ^b^	3.0 ^c^	0.02	<0.001
	*b*	173	166	152	11.10	0.428
	*c*	120 ^b^	152 ^b^	242 ^a^	17.14	0.006
	*PP*	62 ^c^	70 ^b^	97 ^a^	1.08	<0.001

^a, b, c^ Means within a row followed by different letters differ (*p* < 0.05). ^1^ BSA = bovine serum albumin; Rubisco = ribulose 1, 5-disphosphate carboxylase. ^2^ Parameters were obtained by fitting the amount of precipitated protein (mg) and amount of CT (μg) incubated with the equation: *y* = *a_0_ + a*/(1 + exp(−(*x*−*b*)/*c*)), where *y* = mg of protein (BSA or Rubisco) precipitated, *x* = μg of CT incubated, *a_0_ + a* = estimated maximal amount (mg) of protein (BSA or Rubisco) precipitated, *b* = sigmoidal centre (μg of CT at the 50% of maximal protein precipitation), and *c* = sigmoidal width. *PP*: protein-precipitating capacity, expressed as μg CT required to precipitate 1 mg of protein. SEM, standard error of the mean.

**Table 5:** Effects of condensed tannins in purple prairie clover conserved as freeze-dried forage (FD), hay (HAY) or silage (SIL) on the growth of the *Escherichia coli* 25922 and 35218.

Strain	Parameter	Conservation Method	Tannin Concentration (µg/mL)				SEM	*p-*Value
			0	25	50	100		
*E. coli* 25922	*µ*	FD	1.24 ^a^	1.06 ^B,b^	1.06 ^b^	0.91 ^c^	0.032	<0.001
		HAY	1.23 ^a^	1.12 ^A,b^	1.02 ^c^	0.98 ^c^	0.018	<0.001
		SIL	1.25 ^a^	1.15 ^A,b^	1.00 ^c^	0.94 ^c^	0.016	<0.001
		SEM	0.014	0.014	0.03	0.03		
		*p*-value	0.598	0.008	0.368	0.250		
	*L*	FD	0.52 ^b^	0.59 ^A,b^	0.85 ^a^	0.82 ^a^	0.028	<0.001
		HAY	0.53 ^b^	0.49 ^B,b^	0.86 ^a^	0.89 ^a^	0.011	<0.001
		SIL	0.52 ^b^	0.51 ^B,b^	0.82 ^a,b^	0.95 ^a^	0.073	0.006
		SEM	0.004	0.012	0.030	0.085		
		*p*-value	0.422	0.003	0.659	0.570		
*E. coli* 35281	*µ*	FD	1.04 ^a^	0.92 ^b^	0.92 ^b^	0.91 ^b^	0.014	<0.001
		HAY	1.06 ^a^	0.91 ^b^	0.90 ^b^	0.86 ^b^	0.015	<0.001
		SIL	1.05 ^a^	0.89 ^b^	0.91 ^b^	0.91 ^b^	0.016	<0.001
		SEM	0.011	0.018	0.015	0.016		
		*p*-value	0.572	0.583	0.747	0.133		
	*L*	FD	0.32 ^b^	0.32 ^b^	0.33 ^b^	0.41 ^A,a^	0.016	0.011
		HAY	0.32 ^b^	0.30 ^b^	0.32 ^b^	0.39 ^B,a^	0.007	<0.001
		SIL	0.31 ^c^	0.34 ^b,c^	0.35 ^a,b^	0.38 ^B,a^	0.006	<0.001
		SEM	0.008	0.016	0.011	0.005		
		*p*-value	0.530	0.310	0.158	0.032		

Note: The values were obtained by fitting the OD_600_ to the modified Gompertz equation, In (OD_t_/OD_0_) = *A* × exp{−exp ((*µ* × e/*A*) × (*L* − t) + 1)}, where *A* is the logarithmic increase of bacterial population, *µ* is maximum growth rate (per hour), *L* is the lag time, *t* is the time (in hours), and OD_t_ and OD_0_ are the optical densities obtained at time t and at zero hour, respectively. Means with different letters differ (*p* < 0.05). ^A, B^ Capital letters show differences between conservation methods. ^a, b, c^ Lowercase show differences between tannin concentrations. SEM, standard error of the mean.
